# Selective autophagy of non-ubiquitylated targets in plants: looking for cognate receptor/adaptor proteins

**DOI:** 10.3389/fpls.2014.00308

**Published:** 2014-06-25

**Authors:** Vasko Veljanovski, Henri Batoko

**Affiliations:** Institut des Sciences de la Vie, Université Catholique de LouvainLouvain-la-Neuve, Belgium

**Keywords:** selective autophagy cargo receptor, non-ubiquitylated cargo, plant cell, TSPO protein, porphyrins

## Abstract

Cellular homeostasis is essential for the physiology of eukaryotic cells. Eukaryotic cells, including plant cells, utilize two main pathways to adjust the level of cytoplasmic components, namely the proteasomal and the lysosomal/vacuolar pathways. Macroautophagy is a lysosomal/vacuolar pathway which, until recently, was thought to be non-specific and a bulk degradation process. However, selective autophagy which can be activated in the cell under various physiological conditions, involves the specific degradation of defined macromolecules or organelles by a conserved molecular mechanism. For this process to be efficient, the mechanisms underlying the recognition and selection of the cargo to be engulfed by the double membrane autophagosome are critical, and not yet well understood. Ubiquitin (poly-ubiquitin) conjugation to the target appears to be a conserved ligand mechanism in many types of selective autophagy, and defined receptors/adaptors recognizing and regulating the autophagosomal capture of the ubiquitylated target have been characterized. However, non-proteinaceous and non-ubiquitylated cargoes are also selectively degraded by this pathway. This ubiquitin-independent selective autophagic pathway also involves receptor and/or adaptor proteins linking the cargo to the autophagic machinery. Some of these receptor/adaptor proteins including accessory autophagy-related (Atg) and non-Atg proteins have been described in yeast and animal cells but not yet in plants. In this review we discuss the ubiquitin-independent cargo selection mechanisms in selective autophagy degradation of organelles and macromolecules and speculate on potential plant receptor/adaptor proteins.

## DIVERSITY OF TYPES AND TARGETS FOR SELECTIVE AUTOPHAGY

Following his discovery of the lysosome in rat hepatic cells in 1955, the Belgian cytologist and biochemist Christian de Duve, the 1974 Nobel Prize laureate in Physiology or Medicine, coined the term “autophagy” in 1963. This term describes morphologically a process whereby living eukaryotic cells achieve the degradation of their own constituent through a vesicular encapsulation of a portion of their cytoplasm and its degradation in the lysosome/vacuole. Although describing what currently is known as *sensu stricto* non-selective macroautophagy, De Duve and Wattiaux (1966) acknowledged the possibility that this degradation process can be selective, targeting defined cellular structures as opposed to a random and bulk degradation of a portion of the cytoplasm. Macroautophagy (hereafter referred to as autophagy) is an evolutionarily conserved catabolic process allowing eukaryotic cells to recycle nutrients and biosynthetic monomers, and mitigate cellular damage during stressful physiological conditions (Yorimitsu and Klionsky, 2005; Mizushima et al., 2011; Liu and Bassham, 2012). Previously thought to be essentially a non-selective bulk degradation process, within the last decade or so it has been well documented that this complex and highly regulated pathway comes in two main types, non-selective and selective autophagy (Pankiv et al., 2007; Sandoval et al., 2008; Lynch-Day and Klionsky, 2010; Johansen and Lamark, 2011; Levine et al., 2011). One of the distinctive features of autophagy is the *de novo* formation of a double membrane vesicle called an autophagosome, which can fuse with the lysosome/vacuole delivering its content/cargo and membrane constituents for degradation by acidic hydrolases (Mizushima et al., 2011; Klionsky et al., 2012). The formation of an autophagosome is a hierarchical, regulated, and complex series of events involving initiation, elongation, closure, and maturation steps (Thumm et al., 1994; Klionsky et al., 2003; Mizushima et al., 2011; Feng et al., 2014). These steps are marshaled by the coordinated action of four autophagy-related (Atg) protein complexes including a protein kinase complex (Atg1 complex), a lipid kinase complex (Atg6 complex), an ubiquitin-like conjugation complex (Atg5–12–16 complex), and a cycling vesicular complex (Atg9 complex). Out of the 38 Atg gene products described to date in eukaryotic cells, about 15 or so are involved in the activity of these complexes and are known to be conserved through evolution, hence they are considered as core autophagic genes. Both the non-selective and selective autophagic pathways appear to use basically the same core molecular machinery (Behrends et al., 2010; Lamb et al., 2013). Cell type-specific selective autophagy pathways such as (i) the biosynthetic cytoplasm-to-vacuole targeting (Cvt) of defined enzyme precursors in yeast (Klionsky et al., 1992; Lynch-Day and Klionsky, 2010), (ii) the chaperone-mediated autophagy and variants targeting defined motif-containing soluble proteins in mammalian cells, and (iii) various forms of selective microautophagy (Cuervo and Wong, 2014), have not yet been described in plant cells and will not be discussed here. However, selective autophagy is a conserved mechanism in higher eukaryotes, playing a vital physiological role in proteostasis, cell growth, and development. The expanding list of endogenous substrates for this pathway includes organelles such as mitochondria (mitophagy), peroxisomes (pexophagy), chloroplasts (chlorophagy), endoplasmic reticulum (ER; reticulophagy), ribosomes (ribophagy), intracellular pathogens (xenophagy), individual proteins or their aggregates, and non-proteinaceous targets such as lipid droplets or harmful molecules. Selective autophagy is not only critical in the clearance of damaged, superfluous, or dysfunctional cellular structures but also in regulating key molecular mechanisms such as small RNA metabolism, iron utilization by the cell, or antigen presentation to name a few (Crotzer and Blum, 2009; Kirkin et al., 2009; Gibbings et al., 2012; Gump et al., 2013; Mancias et al., 2014). However, the machinery that promotes and regulates selective autophagy is largely unknown, and more so in plant cells for which experimental evidence of this pathway are quite recent and limited.

Selective sequestration and segregation of autophagic targets/cargo from other cellular structures has been observed and characterized both in yeast and mammals. It was only recently that similar observations have been made in plants. Recognition of a given target by a cognate receptor requires a defined ligand or structural feature on the target. A common molecular determinant recognized by selective autophagy receptors in animal cells is conjugated ubiquitin (Rogov et al., 2014). The conjugated ubiquitin on the cargo is bound by receptors of the sequestosome-1-like family such as the modular p62/sequestosome-1 or neighbor of breast cancer 1 (NBR1). Sequestosome-1-like receptors are involved or required for organelles and protein aggregates selective autophagy in mammals (Rogov et al., 2014). Related sequestosome-1-like proteins in plants are functional hybrids (with respect to their modular domains) of both mammalian p62 and NBR1, and may be involved in the clearance of soluble protein aggregates by selective autophagy (Svening et al., 2011; Zientara-Rytter et al., 2011; Zhou et al., 2013). This important family of receptors links the target to the autophagy machinery either directly or via an adaptor protein by interacting with membrane-bound Atg8 family members. However, there is no sequestosome-1-like protein in yeast, and in higher eukaryotes, organelles and other non-proteinaceous targets of selective autophagy are also recognized through other molecular features. In addition, some sequestosome 1-like proteins in mammals such as Optineurin, which is involved in xenophagy, can also target protein aggregates in an ubiquitin-independent manner (Korac et al., 2013).

In addition to the common fundamental and unresolved question of the membrane source for the formation of the corresponding autophagosome, how individual endogenous substrates for selective autophagy are recognized and targeted for degradation is paramount for our understanding of the molecular mechanisms and the biological roles of this pathway. Recent findings suggest that the expanding repertoire of cargo for selective autophagy parallels structurally diverse cognate receptor/adaptor proteins responsible for the recognition and recruitment of the cargo into the autophagosome. We will discuss the recognition and selection processes during mitophagy, pexophagy, and reticulophagy characterized in different cell types including plant cells, and elaborate on the scavenging of porphyrins through the selective autophagic pathway which has also been recently described in plants. Because the molecular mechanism of chloroplast degradation through selective autophagy is not yet clear, we will not discuss chlorophagy.

### MITOPHAGY

Dysfunctional mitochondria are tightly controlled in eukaryotic cells. For instance, oxidative stress within mammalian mitochondria up to a certain level can generate the disposal of oxidized proteins through a vesicular trafficking pathway from the mitochondria to the lysosome (Soubannier et al., 2012; McLelland et al., 2014). This specific pathway is regulated by an E3 ubiquitin ligase, Parkin, and the phosphatase and tensin homologue-induced putative kinase protein 1, PINK1. More damaged mitochondria are degraded by selective autophagy in eukaryotic cells. Parkin and PINK1 are also involved in ubiquitin-dependent selective autophagy of dysfunctional mitochondria. However, selective degradation of dysfunctional mitochondria in yeast is regulated by Atg32, a mitochondrial outer membrane protein (Kanki et al., 2009; Okamoto et al., 2009). Atg32 is a 60 kDa protein anchored to the mitochondrial membrane through a C-terminal transmembrane span. Atg32 connects the mitochondria to the autophagosome machinery by interacting sequentially with the coiled-coil domain-containing adaptor protein Atg11 and the ubiquitin-like protein Atg8. Both Atg11 and Atg32 interact with Atg8 through the so-called Atg8-family interacting motif (AIM, also known as the LC3-interacting region (LIR) in animal proteins). The core consensus of AIM and LIR is W/Y/FxxL/V/I (single amino acid code where x stands for any amino acid; Birgisdottir et al., 2013; Rogov et al., 2014). Structural evidence suggests that the Atg8-like protein contains an aromatic pocket and an aliphatic pocket accommodating the structural determinants of the core AIM/LIR sequence (Noda et al., 2008). It is not yet clear whether Atg32/Atg11 interact with phosphatidylethanolamine-conjugated Atg8 (Atg8-PE, membrane-bound) or soluble Atg8. There is no Atg32 homologue in animal cells, but 3 structurally unrelated mitochondrial outer membrane proteins function as mitophagy receptors. Nix is required for mitochondrial clearance during erythrocyte maturation (Sandoval et al., 2008; Youle and Narendra, 2011) and Bnip3 induces both mitochondria and ER removal by selective autophagy (Zhang et al., 2012). Both proteins are homologous and share the BCL2 homology 3 (BH3) domain and interact with Atg8-family members through an N-terminal LIR (**W**ve**L**). FUN14 domain-containing 1 (FUNDC1) is a mitochondrial outer membrane protein acting as a receptor of hypoxia-induced mitophagy (Liu et al., 2012b). FUNDC1 is membrane-anchored through 3 transmembrane spans and the cytoplasmic N-terminus contains a LIR (**Y**ev**L**). In contrast to Nix and Bnip3, binding of FUNDC1 to Atg8-family members (specifically LC3B) is promoted by phosphorylation of the aromatic residue of the LIR motif (**Y**^P^ev**L**). The kinase responsible for this modification has not yet been identified. Neither Atg32 nor the mammalian mitophagy receptors have homologues in plants. However, an Atg11-related protein (single locus At4g30790) was recently characterized in *Arabidopsis* and elegantly shown to be required for senescence-induced mitophagy in plants (Li et al., 2014). Interestingly, the *Arabidopsis* Atg11-related protein structurally resembles the mammalian RB1-inducible coiled-coil protein 1 (RB1CC1) also known as FIP200 (FAK-family interacting protein of 200 kDa), with an Atg17 functional domain (IPR007240) at the N-terminus and an Atg11 functional domain (IPR19460) at the C-terminus. FIP200 is known to be the functional equivalent of Atg17 in animals, interacting with the Atg1-related kinases and the accessory protein Atg101 during autophagosome formation (Feng et al., 2014). It may be that the structural composition of the Atg1 complex is identical in plant and mammalian cells. Atg17 also acts as an adaptor/scaffold protein during pexophagy in *Pichia pastoris* (Farré et al., 2008). It is not yet clear whether the Atg17 domain in the plant protein is functional. It would be interesting to identify potential interacting partners of the Atg11-domain of the plant protein using mitochondrial outer membrane proteins as bait.

### PEXOPHAGY

The maintenance and turnover of peroxisomes are important for plant development and growth under normal conditions or subjected to (a)biotic stress (Farmer et al., 2013; Hackenberg et al., 2013; Kim et al., 2013; Shibata et al., 2013; Yoshimoto et al., 2014). Marked accumulation of peroxisomes as aggregates were observed in autophagy-deficient *Arabidopsis* mutants (affected in the core autophagy genes; Shibata et al., 2013; Yoshimoto et al., 2014). The peroxisome unusual positioning (Peup) 1, 2, and 4 alleles were shown to be identical to the autophagy core genes Atg2, Atg18a, and Atg7, respectively (Shibata et al., 2013). Interestingly, as compared to control plants, the accumulation of peroxisomes in autophagy-deficient plants was cell type-dependent, since this cellular phenotype was present in leaves but not in roots (Yoshimoto et al., 2014). Further study indicated that the uncleared peroxisomes in leaf cells contained increased levels of catalase, a known substrate for selective autophagy (Yu et al., 2006). In an *Arabidopsis atg5* mutant grown under normal conditions, catalase levels increased eightfold in leaves but the catalase levels remained unaffected in root tissues. In plants, it seems that catalase acts upstream of immunity-triggered autophagy. The reaction of catalase with reactive oxygen species (ROS) allows catalase to act as a molecular link between ROS and the promotion of autophagy-dependent death (Hackenberg et al., 2013; Shibata et al., 2013). Intriguingly, isolated leaf peroxisomes do not show a substantial increase in ubiquitylated proteins after immuno-electron microscopy using anti-ubiquitin, suggesting that the aggregated peroxisomes are not cleared by an ubiquitin-dependent mechanism (Yoshimoto et al., 2014). Atg36 and Atg30 are receptors for pexophagy in *Saccharomyces cerevisiae* and *P. pastoris*, respectively (Farré et al., 2013). Both proteins are recruited to peroxisomes by the peroxisomal membrane protein Pex3, and contain AIM and bind to Atg8-family members. Phosphorylation of the pexophagy receptor proteins at the vicinity of their AIM also regulates their interaction with Atg11 or Atg17, and enhances their affinity for Atg8 (Farré et al., 2013). To date, no related counterparts of Atg36 or Atg30 have been described in plants.

### RETICULOPHAGY

Secreted proteins are synthesized in the ER and this organelle is also the site of stringent quality control for the correct folding and quaternary structure of proteins in eukaryotic cells. For instance, misfolded polytopic membrane proteins are targeted for ER-associated degradation (ERAD). Protein misfolding can arise for example as a consequence of cellular stress or mutation in the primary structure of the polypeptide. A recent work in mammalian cells showed that the ER-associated HSP40 chaperone JB12 participates in partitioning mutant conformers of the gonadotropin releasing hormone receptor (GnRHR), a G-protein-coupled receptor, between ERAD and what was coined the ER quality control autophagy pathway (Houck et al., 2014). This selective autophagy pathway degrades the E90K-GnRHR mutant form which is ERAD-resistant. Interaction between ER-associated HSP40s and the vacuolar protein sorting 34 complex may allow the selective autophagy degradation of ERAD-resistant membrane proteins from the ER (Houck et al., 2014).

Experimental evidence was provided recently that plant cells also use reticulophagy as a response to ER stress (Liu et al., 2012a). Chemical ER stress agents-triggered reticulophagy requires the ER stress sensor inositol-requiring enzyme-1b (IRE1b).

In addition, an ER-to-vacuole pathway was also described in plant cells, and this pathway is regulated by the putative cargo receptors *Arabidopsis thaliana* Atg8-interacting proteins ATI1 and ATI2, which are unique to plants (Honig et al., 2012). ATI1 and ATI2 bind Atg8-family members via their conserved AIM **W**qv**L**, and could help segregate specific cargo molecules from the ER for their transport and degradation in the vacuole, although none has been identified yet.

### HEME AND AtTSPO DETOXIFICATION

**FIGURE 1:**
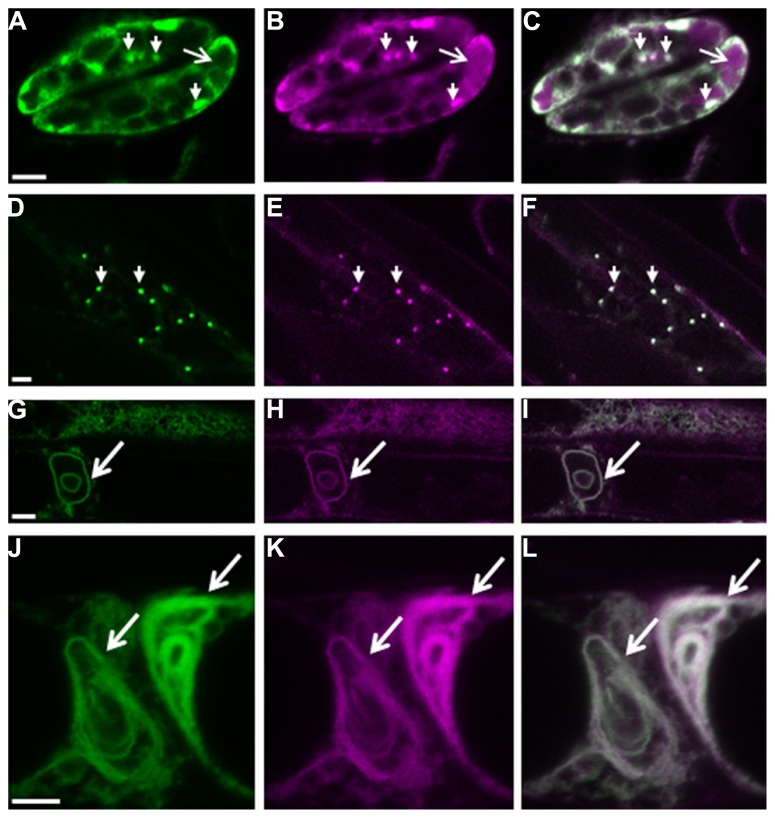
**Autophagy-dependent degradation of AtTSPO**. Confocal images of a mCherry-GFP-AtTSPO fusion stably expressed constitutively in transgenic *Arabidopsis thaliana*; the green signal represents GFP fluorescence, magenta mCherry fluorescence, and the overlapping signal is shown as white in the merge images. Arrowheads in panels **A–C** indicate Golgi stacks in guard cells, and the arrow highlights mCherry fluorescence (but not GFP) in the vacuole. Panels **D–F** show imaging of the fluorescent chimera in a root cell, and the arrowheads indicate Golgi stacks. Panels **G–I** show the formation of autophagy bodies (arrow) in root cells after Concanamycin A treatment. Panels **J–L** show complex entangled membranes of autophagy bodies after Concanamycin A treatment. Scale bar is 5 μm in **A–I**, and 2 μm in **J–L**.

We recently showed that plant cells can scavenge toxic free heme in the cytoplasm through the autophagic pathway (Vanhee et al., 2011). In contrast to other higher eukaryotes, heme biosynthesis, and heme oxygenase (ER-localized in mammalian cells) which is responsible for heme degradation, are localized in plant plastids. Abiotic stress transiently up-regulates heme biosynthesis (required for the activity of ROS scavengers) and simultaneously induces the expression of the polytopic TSPO-related membrane protein AtTSPO (*Arabidopsis thaliana* Translocator-related protein). Because heme functions not only as a prosthetic group for a myriad of proteins scattered throughout the cell but also as a regulatory signaling molecule (Severance and Hamza, 2009), this suggests that “free” heme is present albeit transiently in the cell. We showed that the ER-Golgi-localized AtTSPO (Guillaumot et al., 2009) binds heme *in vivo* and the complex is recruited to the autophagosome through an AIM present in AtTSPO (Vanhee et al., 2011). Constitutive expression of AtTSPO is toxic to plant and yeast (*S. cerevisiae*, devoid of TSPO-related protein) cells. When expressed as a fluorescent fusion, AtTSPO accumulates in autophagic bodies after concanamycin A treatment (**Figure 1**), suggesting an active degradation of the protein through the autophagic pathway. Mammalian TSPO proteins are encoded by essential genes indicating a functional divergence with seed plant TSPOs. AtTSPO can oligomerize and seems to function as an authentic autophagy receptor at least for toxic porphyrins in the plant cell.

## FORMATION AND MATURATION OF SELECTIVE AUTOPHAGOSOMES IN PLANT CELLS

**FIGURE 2:**
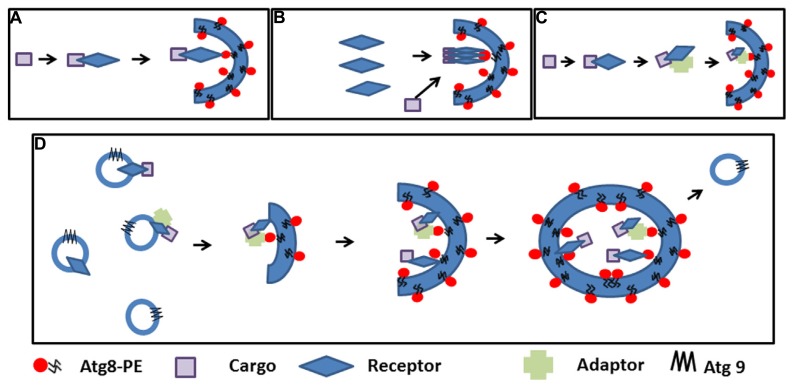
**Cargo selection and speculative autophagosome formation during selective autophagy**. **(A)**. The cargo is recognized by the receptor and the complex recruits pre-existing phagophores by receptor-Atg8 interaction. **(B)**. First polymeric autophagy receptors are recruited in the phagophore through Atg8 interaction followed by the cargo. **(C)**. The cargo is recognized by the receptor and the complex binds an adaptor before recruiting pre-existing phagophores though Atg8 interactions. **(D)**. Interaction between the receptor, the cargo (with or without an adaptor protein) nucleate the initiation of the phagophore and recruit the autophagy machinery; elongation of the phagophore and concentration of the cargo is achieved through Atg9-containing cycling vesicles

Where and how selective autophagosomes are initiated in the plant cell is still not clear. Hypothetically, the autophagy machinery could be recruited on the cargo by the receptor-Atg8 complex to promote phagophore nucleation. The elongated double membrane could then close and engulf the cargo. Alternatively, the receptor-cargo complex could recruit a preformed phagophore/initial membrane (**Figure 2A**). The latter supposes the presence of preformed phagophores, which are probably initiated to sustain basic non-specific autophagy (Liu and Bassham, 2012). Autophagosomes can be rapidly induced in the cell; therefore, the cell must have the capacity to mobilize a substantial amount of membrane to create these double membrane structures (Lamb et al., 2013). Many studies in mammalian cells and in yeast to some extent favor the ER and ER-mitochondrial contact sites as the most common origin of autophagosomal membranes, although other evidence suggests that several cellular compartments including the Golgi, the plasma membrane, endosome, and mitochondria contribute to the expansion of the nascent phagophore (Lamb et al., 2013; Puri et al., 2013). Recent molecular evidence also points to the ER as the structure initiating autophagosomes in the plant cell (Zhuang et al., 2013). ER-bound selective autophagy receptors such ATI1 and ATI2 could nucleate the formation of specialized autophagosomes after interacting with Atg8-PE and contributing to the assembly of molecular platforms on which the selective autophagosomes are formed. In yeast, Atg8-PE conjugation to various membranes occurs constantly and independently of autophagy-inducing stimuli (Kirisako et al., 2000; Nair et al., 2012). Recent evidence suggests that Atg-family members recruited by selective autophagy receptors to the cargo directly engage regulatory and core autophagy proteins, potentially priming the site of autophagosome formation (Rogov et al., 2014). Concentration of the cargo may require oligomerization of the receptor, and adaptor or scaffold protein-containing AIM may help bridge the complex to membrane-bound Atg8 (Figures 2B,C). The elongation step is then regulated in part by cycling Atg9-containing vesicles that supply the required membrane lipids, but also additional receptor-cargo complexes and regulatory components (Lamb et al., 2013; **Figure 2D**). The subcellular localization and trafficking of the polytopic membrane protein Atg9 are not yet known in plants. It is tempting to speculate that membrane bound receptors such as AtTSPO linked to their cargo could be enriched in the autophagosomal membrane through vesicular transport mediated by Atg9.

## CONCLUDING REMARKS AND PERSPECTIVES

The repertoire of targets for selective autophagy is expanding quite fast, and so is the list of individual cognate receptor/adaptor involved in their recognition. The mechanism of recognition involves specific ligands (such as ubiquitin or others) or structural patterns on the target. The receptor proteins characterized so far suggest non-overlapping roles of the cargo receptors for the same target and an intriguing lack of evolutionary conservation, complicating the *in silico* search for potential candidates. A functional AIM is not an indication of potential cargo receptor function, as a plethora of proteins including autophagic substrates and regulators contain AIMs. Some cargo receptors also contain functional atypical AIM/LIR without the canonical aromatic residue (von Muhlinen et al., 2012). In addition, Atg5 plays a crucial role in selective autophagosome formation and Atg5-binding proteins are emerging selective autophagy receptors.

## Conflict of Interest Statement

The authors declare that the research was conducted in the absence of any commercial or financial relationship that could be construed as a potential conflict of interest.
